# Grifolin exhibits anti-cancer activity by inhibiting the development and invasion of gastric tumor cells

**DOI:** 10.18632/oncotarget.15250

**Published:** 2017-02-10

**Authors:** Zijian Wu, Ying Li

**Affiliations:** ^1^ Tianjin Key Laboratory of Food Biotechnology, College of Biotechnology and Food Science, Tianjin University of Commerce, Tianjin, China; ^2^ Tianjin Neurological Institute, Tianjin Medical University General Hospital, Tianjin, China

**Keywords:** gastric cancer, grifolin, natural anti-cancer product, apoptosis, cancer invasion

## Abstract

Grifolin, a natural product isolated from the mushroom *Albatrellus confluens*, has been reported to be a potent anti-cancer agent in nasopharyngeal carcinoma and osteosarcoma. The data obtained in this study revealed that grifolin is capable of inhibiting the growth and invasion of gastric cancer cells by inducing apoptosis and suppressing the ERK1/2 pathway. Our results support the potent utility of grifolin as an anti-tumor lead compound against gastric cancer cells.

## INTRODUCTION

Gastric cancer (GC) is one of the main causes of cancer-associated mortality, particularly in East Asia, where it has an incidence of approximately 934,000 cases per year [1]. Surgical resection and chemotherapy are still the two major approaches for treating gastric cancer [2], although its recurrence and metastasis are still obstacles for combination chemotherapies [3].

Grifolin, a secondary metabolite isolated from the mushroom *Albatrellus confluens*, is capable of inhibiting the growth of cancer cells *in vitro* by inducing the apoptotic pathway [4] with both high efficiency and low toxicity [5]. However, the mechanisms of grifolin in tumor cells are not completely understood, although grifolin evidently possesses multiple biological activities, including the inhibition of histamine release and NO production [6], antibiotic [7] and anti-oxidant [8] properties and a plasma cholesterol-lowering effect. It was also suggested that the death-associated protein kinase 1 (*dapk1*) gene, which is an apoptosis-related gene, was upregulated at least twofold in response to grifolin treatment in the nasopharyngeal carcinoma cell line CNE1. In brief, grifolin plays an important role in treating nasopharyngeal carcinoma [7] and osteosarcoma [8] by serving as a potent anti-cancer agent. However, it has not yet been reported whether it also possesses an antitumor effect in gastric cancer. Herein, we investigated the functions of grifolin in gastric cancer.

Our results showed that grifolin had a significant antitumor effect that was achieved by regulating cellular proliferation, inducing apoptosis and inhibiting human gastric cancer invasion. Our finding provides a deep insight into the anti-tumor mechanisms of grifolin and will provide a theoretical basis for the clinical treatment of gastric cancer.

## RESULTS

### Expression profiles of genes reveal the potential antitumor effect of grifolin in gastric cancer cells

**Figure 1:**
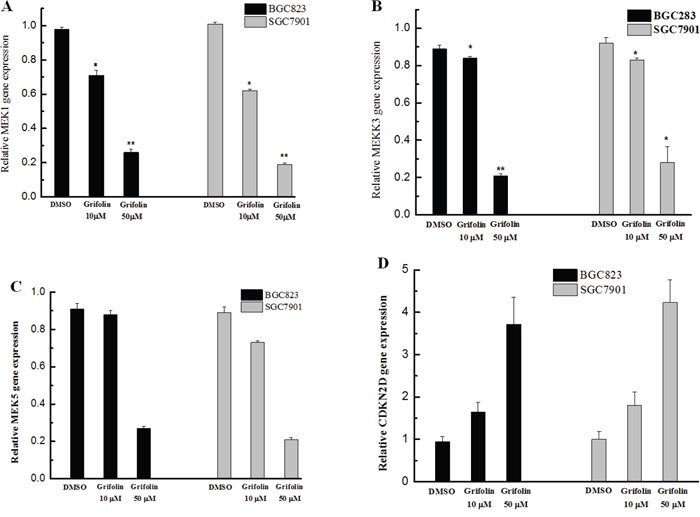
Effects of grifolin on gene expression in GC cells The expression of genes in BGC823 and SGC7901 cells treated with grifolin (10 and 50 μM) was detected using qRT-PCR. Data are at least three independent experiments and are shown as the mean ± SD (*P<0.05, **P<0.01, ***P<0.001).

The expression levels of related genes in the MAPK signal pathway were determined using qRT-PCR after treatment with grifolin. From the result, it was observed that MEK1 expression was markedly decreased upon treatment with grifolin (Figure 1A), whereas a significant decrease in both MEKK3 and MEK5 expression was observed only in cells treated with a high dose of grifolin (50 μM) (Figure 1B, 1C). Our finding indicated that grifolin might inhibit GC metastasis by modulating the MAPK pathway. In addition, the cyclin-dependent kinase 4 inhibitor 2D (CDKN2D), which is a cell-cycle regulator, was significantly upregulated, thus suggesting the potent regulatory activity of grifolin on the cell cycle (Figure 1D).

### Grifolin suppresses cell invasion

**Figure 2:**
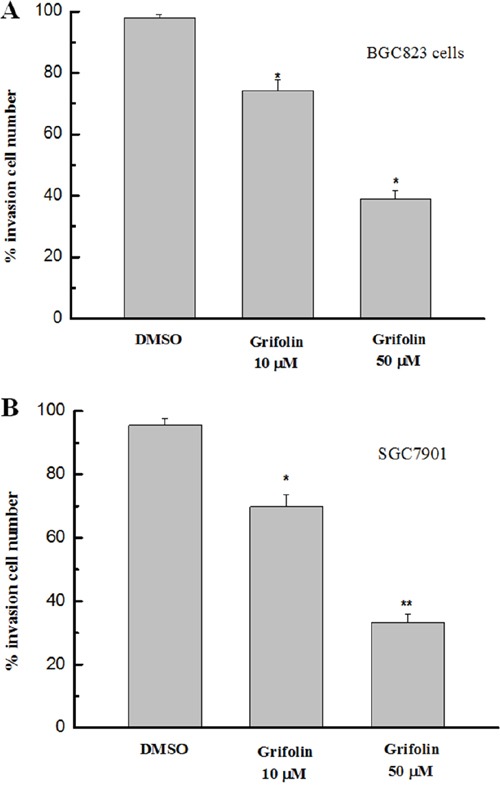
Effects of grifolin on cell invasion The invasiveness of GC cells treated with grifolin was measured by transwell assay. The results indicated that a significant decrease in cell invasion followed the administration of grifolin. Data are from at least three independent experiments and are shown as the mean ± SD (*P<0.05).

We further investigated the potential anti-metastatic activity of grifolin in gastric cancer cells using the transwell assay. As shown in Figure 2, the invasiveness of BGC823 and SGC7901 cells was significantly suppressed following treatment with grifolin, which indicated that grifolin might inhibit the invasion of gastric cancer cells.

### Cell cycle arrest assay

**Figure 3:**
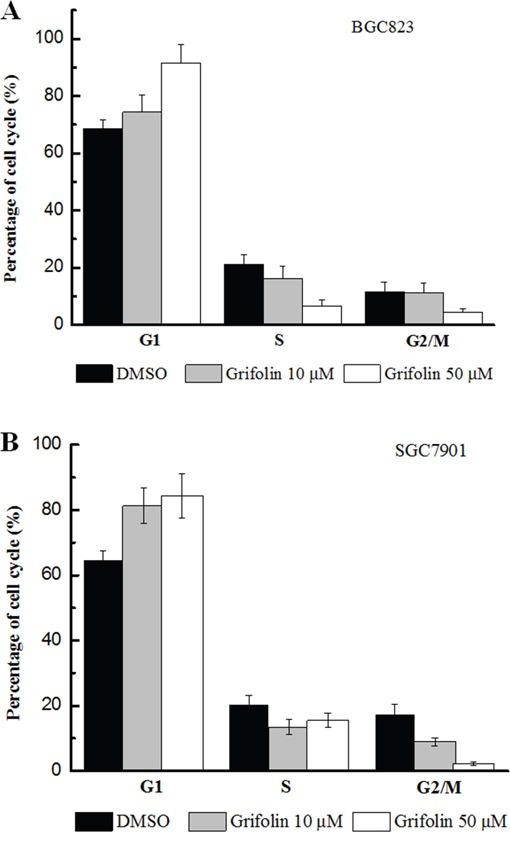
Effects of grifolin on cell cycle **Panel A**. Effect of grifolin in cell cycle of BGC823. **Panel B**. Effect of grifolin in cell cycle of SGC7901. The treated BGC823 and SGC7901 cells were collected, and the cell cycle distribution was analyzed with using flow cytometer following PI staining. The results of the cell cycle assays also indicated that grifolin caused an accumulation of cells in G1. The percentage of G1 cells increased from approximately 65% in the control to approximately 92% in populations treated with 50 μM grifolin in both BGC823 and SGC7901 cells; consequently, fewer cells progressed to the S phase, ranging from approximately 25% in the untreated group to approximately 3.5% in the treated group, and the percentage of G2/M cells decreased from approximately 15% to approximately 5%.

The cell cycle assays described in this study indicated that grifolin caused an accumulation of tumor cells in the G1 phase. The percentage of G1 cells increased from approximately 65% to 92% in both the BGC823 and SGC7901 cell populations after treatment with grifolin (50 μM); consequently, fewer cells progressed to the S and G2/M phases. This finding indicated that cell cycle progression was dramatically blocked in G1 when GC cells were treated with grifolin (Figure 3A, 3B).

### Apoptotic assay

**Figure 4:**
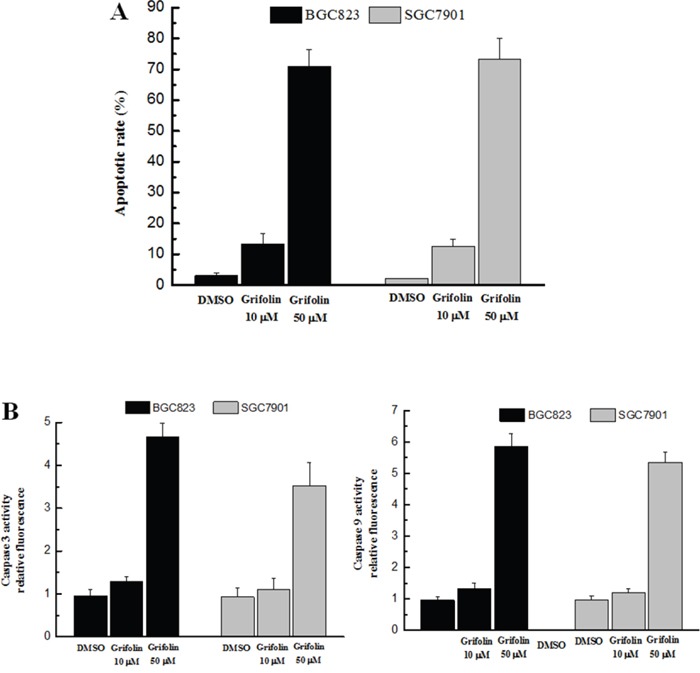
Effect of grifolin on cell apoptosis **Panel A**. The apoptosis rate in treated BGC823 and SGC7901 cells was examined using flow cytometric analysis. **Panel B**. The activation of caspase 3 (left) and caspase 9 (right) was determined using the caspase-3/9 fluorescent assay. The results indicate that the apoptosis rates of both BGC823 and SGC7901 cells were dramatically increased after treatment with grifolin following the activation of caspase 3 and caspase 9.

The apoptotic assay performed in this study indicated that the apoptosis of both BGC823 and SGC7901 cells was dramatically increased with the presence of grifolin (Figure 4A). In addition, the activation of caspase 3 and caspase 9 was significantly increased by grifolin (Figure 4B). The data suggested that grifolin induced apoptosis in gastric cancer cells that was consistent with its anti-tumor activity.

### Anti-tumor efficacy of grifolin in human tumor xenograft mice

**Figure 5:**
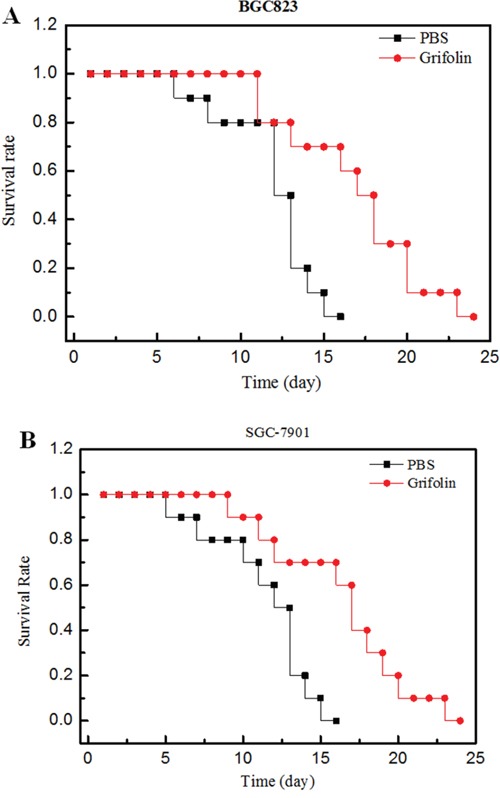
Anti-tumor efficacy of grifolin on human tumor bearing mice **Panel A**: Effect of conjugate on human xenograft tumor model mice bearing BGC823 cells. **Panel B**: Effect of conjugate on human xenograft tumor model mice bearing SGC-7901 cells. **Legend**: The results indicate that grifolin (●) produced an improved survival time compared to PBS (■) in BGC823 (Panel A) and SGC-7901 (Panel B) bearing mice, p<0.05 (n=10). **Condition**: Xenograft mice bearing BGC823 and SGC-7901 cells were treated with grifolin. Survival time was recorded in days after the injection. All data obtained from repeated experiments were pooled and utilized for statistical analysis.

To evaluate the anti-tumor effect of conjugated and free paclitaxel, mice with tumors derived from either BGC823 or SGC-7901 cells were treated with grifolin (15 mg/kg body weight/2 day). The antitumor activity was determined by monitoring the animal survival rate over 30 days of treatment. The results shown in Figure 5 reveal that the administration of grifolin significantly increased the survival rate of experimental mice.

## DISCUSSION

Gastric cancer, one of the most common primary malignancies, is still a medical challenge to public health. Clinical treatments for gastric cancer, which include surgical resection, embolization, ablation, and chemotherapy, are limited by toxicity and side effects [2]. Therefore, these unmet clinical needs have initiated a growing number of studies aiming to find novel antitumor drugs. Thus, many natural products and their dietary constituents have attracted attention because of their potent anti-tumor properties and lower toxicity. In this study, the results indicate that grifolin may be able to inhibit the growth of GC cells *in vitro* by inhibiting cell proliferation, suppressing cell invasion, and promoting cell apoptosis.

Grifolin also seemed to suppress cell proliferation and regulate the cell cycle in GC cells via the ERK1/2 pathway but not the ERK5 pathway because the ERK5 pathway showed less sensitivity to grifolin than did the ERK1/2 pathway. The ERK1/2 and BMK1/ERK5 pathways play key roles in the regulation of multiple biological activities, including cell proliferation, differentiation, cell cycle transition, and survival [9]. ERK1/2 can be activated by MAPK/ERK kinase 1/2 (MEK1/2) [9], whereas ERK5 (BMK1), a recently identified member of the mammalian MAPK family, is activated not by MEK1 or MEK2 but by MEK5 [10]. The ERK1/2 pathway can regulate the expression of cyclin D1, which is responsible for the G1/S transition [11]. Inhibiting the ERK1/2 pathway blocks the proliferation of many cell types in the G1 phase [12–14]. Similarly, ERK5 is required for the G1-to-S cell cycle transition, and decreased ERK5 expression inhibits the proliferation and arrests the cell cycle in G1 [15]. It is confirmed that the constitutive activation of the ERK1/2 pathway contributes to tumorigenesis, or cancer growth, and increases the cell death threshold [16]. According to our findings, it was presumed that grifolin could suppress cell proliferation and the cell cycle mainly by inhibiting the phosphorylation and kinase activity of ERK1/2 but not that of ERK5 [16].

In combination with caspase-dependent apoptosis induced by grifolin, our evidence indicates that grifolin can effectively inhibit cell proliferation and invasion and induce apoptosis in gastric cancer cells. This is the first study to demonstrate the potential anti-cancer effect of grifolin in GC cells, which might be a novel agent or lead compound for the clinical treatment of gastric cancer.

## MATERIALS AND METHODS

### Materials

The human gastric cancer cell lines BGC823 and SGC-7901 were purchased from the Cell Bank of the Shanghai Institutes of Chinese Academy of Sciences. Grifolin (2-trans, trans-farnesyl-5-methylresorcinol) was provided by the Kunming Institute of Botany, Chinese Academy of Sciences (purity >99%, HPLC analysis).

### Methyl thiazolyl tetrazolium (MTT) assay

BGC823 and SGC7901 cells were seeded in 96-well plates at a density of 5 × 10^4^ cells per well, allowed to adhere overnight, and then treated with grifolin as described above. Cell viability was analyzed using an MTT assay (Sigma, MO) at the indicated time points, according to the manufacturer's instructions. In brief, 1 μl/well of MTT was added and the cells were incubated at 37°C for an additional 4 h. Then, the medium was discarded and the cells were lysed in DMSO (150 μl/well). The absorbance at 490 nm was measured on a plate reader. Each experiment was performed in triplicate and repeated three times.

### q-RT PCR assay

BGC823 and SGC-7901 cells were plated in 6-well plates and then incubated with grifolin at final concentrations of 10 μM and 50 μM for 48 hours, respectively. Total RNA was extracted from BGC823 and SGC7901 cells using Trizol reagent, followed by further purification and analysis with the Agilent Bioanalyzer 2100. Quantitative real-time PCR (qRT-PCR) of the genes MEK1, MEKK3, MEK5, CDKN2D and GAPDH was performed using SYBR Premix ExTaqTM II kit (TaKaRa, Dalian, China). The conditions of the qRT-PCR were as follows: 94°C for 10 s, 94°C for 5 s, 52°C for 30 s to anneal, and 72°C for 15 s for 40 cycles, with detection at 62°C. PCR amplifications were performed with three duplicates for each sample. The relative RNA expression was calculated using the 2-ΔCt method. The specific primers sequences are listed in Table 1.

**Table 1:** List of primers

Primer	Sequence (5′-3′)
**MEK1-assay-F**	**TGCCAGGCTGAACTACAGTA**
**MEK1-assay-R**	**CACAAGGCTCCCTCTCAGAC**
**MEKK3-assay-F**	**GATGGCAGAAGAACATTT**
**MEKK3-assay-R**	**ACCCATGTTCTCGCCATT**
**MEK5-assay-F**	**ATGCTGTGGCTAGCCCTTGG**
**MEK5-assay-R**	**GTAATATCTAGTAGTATGACC**
**CDKN2D-assay-F**	**GCCTTGCAGGTCATGATGTTTGGA**
**CDKN2D-assay-R**	**ATTCAGGAGCTAGGAAGCTGACCA**
**GAPDH-assay-F**	**CATCACCATCTTCCAGGAGCG**
**GAPDH-assay-R**	**TGACCTTGCCCACAGCCTT**

### Cellular invasion assay

The inhibitory effect of grifolin against the invasion of gastric cancer cells was studied using a transwell assay in a Biocoat Matrigel Invasion Chamber. The membranes were fixed in buffered formalin and stained with crystal violet before counting under a microscope in five randomly selected fields.

### Cell cycle arrest

An appropriate number of cells, as described previously, was collected, washed, suspended in PBS and fixed in 75% ethanol. The fixed cells were stained with propidium iodide (PI) supplemented with RNaseA (Sigma) and analyzed using a FACScan flow cytometer (BD Biosciences). Data were collected and analyzed using the ModFit software (BD Biosciences).

### Detection of apoptotic properties

To detect apoptosis, an appropriate number of adherent cells was collected, washed and suspended in cold PBS for analysis. Apoptosis was detected using the Alexa Fluor^®^ 647/7-AAD apoptosis kit (BioLegend) according to the manufacturer's recommended instructions. Data were assessed by flow cytometry (BD Biosciences).

The activation of caspase-3/9 was investigated in this assay as an important index. The cells were treated with grifolin and then resuspended in lysis buffer (pH 7.5, 25 mM HEPES, 5 mM MgCl2, 5 mM EDTA, 5 mM DTT, 2 mM PMSF, 10 mg/ml pepstatin A and 10 mg/ml leupeptin). Cell lysates were centrifuged at 12,000 g for 5 min, and supernatants containing 50 mg of protein were incubated with 50 mM Ac-DEVD-AMC (substrate for caspase-3) or Ac-LEHD-AMC (substrate for caspase-9) at 37°C for 1 h. The fluorescence of AMC was measured using a spectrofluorometer (Hitachi F-4500), with excitation at 360 nm and emission at 460 nm (Promega CaspACE2 Assay System).

### Treatment of human tumor xenograft mice

Six-week-old male BALB/c nude mice were obtained from the Shanghai Laboratory Animal Co., China Academy of Sciences (Shanghai, China). Mice weighing 16-20 mg were housed in barrier facilities on a 12-h light/dark cycle. Food and water were supplied *ad libitum*. On day zero, two groups of mice were inoculated via i.p. injection with BGC823 and SGC-7901 tumor cells (2× 10^5^) suspended in 0.5 ml of RPMI1640. The treatment was initiated after the tumor was allowed to grow to approximately 100 mm^3^ on the back of the mouse. This experimental protocol was intended to mimic the clinical situation, where treatment begins after a tumor has already been established in the treatment of human tumor xenograft mice. The animals were treated with grifolin at the specified dose (15 mg/kg body weight) every 2 days. Control groups were treated with PBS. During an experimental period of 30 days, the survival ratio was recorded as the number of days after tumor injection. The mean and median survival times and the statistical significance of the results were determined using a two-tailed Wilcoxon's ranking test.

### Statistical analysis

All experiments were performed independently at least three times. All data are presented as the mean values ± standard deviations (SD) of each group. Statistically significant differences were calculated by two-tailed Student's t-test using the SPSS software (version 19.0). The graphs were generated with Origin 7.0.
